# 2563. Cytomegalovirus (CMV) Prophylaxis with Letermovir (LET) in Pediatric (Birth to < 18 years of age) Allogeneic Hematopoietic Cell Transplant (HCT) Recipients: Pharmacokinetics (PK), Safety and Efficacy Results of a Phase 2b study

**DOI:** 10.1093/ofid/ofad500.2180

**Published:** 2023-11-27

**Authors:** Johannes H Schulte, Aharon Gefen, Andreas H Groll, Christopher J Fraser, Valerie L Teal, Barbara A Haber, Christopher L Gilbert, Jacqueline B McCrea, Mayankbhai Patel, Karsten Menzel, Cyrus Badshah

**Affiliations:** Charité Universitätsmedizin Berlin, Berlin, Berlin, Germany; Ruth Rappaport Children’s Hospital, Haifa, Tel Aviv, Israel; University Children’s Hospital Muenster, Muenster, Germany, Muenster, Nordrhein-Westfalen, Germany; Queensland Children’s Hospital, Brisbane, Queensland, Australia; Merck & Co., Inc., Rahway, New Jersey; Merck & Co., Inc., Rahway, New Jersey; Merck & Co., Inc., Rahway, New Jersey; Merck & Co., Inc., Rahway, New Jersey; Merck & Co., Inc., Rahway, New Jersey; Merck & Co., Inc., Rahway, New Jersey; Merck & Co., Inc., Rahway, New Jersey

## Abstract

**Background:**

LET is a CMV DNA terminase complex inhibitor approved for prophylaxis of CMV infection and disease in adult CMV-seropositive allogeneic HCT recipients. This study evaluated PK, safety and efficacy of LET for CMV prophylaxis in pediatric allogeneic HCT recipients.

**Methods:**

This was a Phase 2b, single-arm, multicenter, open-label study (NCT03940586). Participants (pts) from birth to < 18 years (y) were sequentially enrolled in 3 age groups (AG): AG1, 12 to < 18 y; AG2, 2 to < 12 y; AG3 < 2 y. All pts received LET orally (PO) or intravenously (IV) with/without cyclosporin A (CsA). Pediatric target exposures were based on model-predicted Phase 3 population simulations for adult HCT recipients, ranging from 16,900 ng.h/mL (480 mg PO, no CsA) to 147,800 ng.h/mL (480 mg IV, no CsA). PK parameters were determined by non-compartmental analysis (5 samples over 24 hr, 38/63 pts). Safety was assessed by evaluation of adverse events.

**Results:**

Of 63 pts administered LET, the majority were White (69.8%) and male (69.8%); median age of 11 (0–17) y; median weight of 32.4 (5.1–95.0) kg.

PK (n=36 evaluable pts): AG1 (n=12, LET 480 mg; LET 240 mg + CsA) achieved exposures within safety margins of adult HCT pts. AG2 (n=16, LET 60–240 mg PO/IV; LET 60–120 mg IV + CsA): 14 pts (PO n=6, IV n=8) who received LET alone achieved target range exposures; 2 pts who received IV LET with CsA had exposures slightly lower than target range. AG3 (n=8, LET 40 mg IV; 60 mg PO; LET 20 mg IV + CsA; 40 mg PO; 60 mg PO/IV): 3 pts who received LET with CsA achieved exposures trending lower than median target. Therefore, LET dose was increased for 5 pts (< 10 kg and aged < 2 y) to achieve target range exposures.

Safety (n=63): LET was well-tolerated with a safety profile similar to that observed in adults.

Efficacy (n=56): The proportion of pts with clinically significant CMV infection (CMV disease/pre-emptive treatment for CMV viremia) through Week 24 post-HCT was similar (25%) to that seen in adults receiving LET in the Phase 3 study (37.5%).

**Conclusion:**

LET administration for CMV prophylaxis in pediatric HCT recipients at doses used in this study resulted in exposures within development program safety margins and was associated with similar safety and efficacy when compared with LET use in adult HCT recipients.

**Disclosures:**

**Johannes H. Schulte, MD**, The German Cancer Consortium (DKTK): Grant/Research Support **Andreas H. Groll, MD**, Amplyx: Advisor/Consultant|Astellas: Advisor/Consultant|Astellas: sered at the speakers’ bureau|Basilea: Advisor/Consultant|Basilea: served at the speakers’ bureau|F2G: Advisor/Consultant|F2G: served at the speakers’ bureau|Gilead Sciences: Advisor/Consultant|Gilead Sciences: Grant/Research Support|Gilead Sciences: served at the speakers’ bureau|Merck Sharp & Dohme LLC: Advisor/Consultant|Merck Sharp & Dohme LLC: Grant/Research Support|Merck Sharp & Dohme LLC: served at the speakers’ bureau|Pfizer: Advisor/Consultant|Pfizer: Grant/Research Support|Pfizer: served at the speakers’ bureau **Valerie L. Teal, MS**, Merck Sharp & Dohme LLC: Wages and Salary made to Author|Merck Sharp & Dohme LLC: Stocks/Bonds **Barbara A. Haber, MD**, Merck Sharp & Dohme LLC: Wages and Salary made to Author|Merck Sharp & Dohme LLC: Stocks/Bonds **Christopher L. Gilbert, n/a**, Merck Sharp & Dohme LLC: Wages and Salary made to Author|Merck Sharp & Dohme LLC: Stocks/Bonds **Jacqueline B. McCrea, PharmD**, Merck Sharp & Dohme LLC: Wages and Salary made to Author|Merck Sharp & Dohme LLC: Stocks/Bonds **Mayankbhai Patel, PhD**, Merck Sharp & Dohme LLC: Wages and Salary made to Author|Merck Sharp & Dohme LLC: Stocks/Bonds **Karsten Menzel, PhD**, Merck Sharp & Dohme LLC: Wages and Salary made to Author|Merck Sharp & Dohme LLC: Stocks/Bonds **Cyrus Badshah, MD, PhD**, Merck Sharp & Dohme LLC: Wages and Salary made to Author|Merck Sharp & Dohme LLC: Stocks/Bonds

